# Efficacy and safety of epsilon-aminohexanoic acid and tranexamic acid during posterior interbody fusion surgery

**DOI:** 10.3389/fsurg.2025.1661609

**Published:** 2025-11-10

**Authors:** Dilixiati Ainiwaer, Ayinuer Tuersun, Jiang Li, Xiaomei Li

**Affiliations:** 1 Department of Pharmacy, Xinjiang Uygur Autonomous Region Sixth People's Hospital, Urumqi, Xinjiang, China; 2 The Sixth Affiliated Hospital of Xinjiang Medical University, Urumqi, Xinjiang, China

**Keywords:** lumbar disc herniation, lumbar spondylolisthesis, lumbar spinal stenosis, posterior lumbar interbody fusion, tranexamic acid, epsilon-aminohexanoic acid, blood loss

## Abstract

**Background:**

Tranexamic acid (TXA) is a proven effective and favored antifibrinolytic hemostatic drug, while epsilon-aminocaproic acid (EACA) has only recently been applied in the field of orthopedics. Few studies compare the efficacy of these two drugs in spinal surgery. We evaluated the hemostatic performance and safety of aminocaproic acid, and explored whether aminocaproic acid can be used as a substitute for TXA during posterior lumbar interbody fusion (PLIF) surgery, providing theoretical support for the flexible selection of hemostatic drugs during spinal surgery.

**Methods:**

We conducted retrospective analysis of 180 patients with lumbar disc herniation, lumbar spinal stenosis, and lumbar spondylolisthesis, who had been admitted to the spinal surgery department of the Our hospital or The Sixth Affiliated Hospital of Xinjiang Medical University, between September 2021 and May 2023, and underwent PLIF. According to the types of hemostatic drugs used during the perioperative period, the patients were divided into two groups, namely, the EACA group (*n* = 86) and the TXA group (*n* = 94). The main outcome measures were total blood loss, total red blood cell loss, and transfusion volume/rate. Other outcome measures included length of hospital stay, hospitalization costs, deep vein thrombosis rate, and biochemical hematological indicators, specifically indicators related to anemia, nutrition, and coagulation.

**Results:**

(1) The red blood cell width of the EACA group (43.94 ± 10.56) was significantly higher than that of the TXA group (40.45 ± 12.54), with a statistically significant difference (*p* < 0.05). (2) The postoperative total protein of the EACA group (56.17 ± 7.83) was significantly lower than that of the TXA group (59.3628 ± 6.73), with a statistically significant difference (*p* < 0.05). (3) The postoperative international normalized ratio of the EACA group (1.06 ± 0.14) was significantly lower than that of the TXA group (1.14 ± 0.13), with a statistically significant difference (*p* < 0.05). There was no statistically significant difference between the two groups in terms of other indicators.

**Conclusion:**

There was no significant difference in total blood loss, total red blood cell loss, transfusion volume/rate, postoperative hospitalization time, hospitalization costs, and surgical complications between the intravenous EACA and TXA groups during PLIF surgery. The two groups had similar hemostatic effects and safety outcomes. Therefore, when selecting antifibrinolytic drugs during PLIF surgery, EACA can be considered an alternative to TXA. However, large-scale, multicenter randomized controlled studies are still required to gauge its later-stage efficacy.

## Introduction

Posterior lumbar interbody fusion (PLIF) is a surgical method for the treatment of lumbar spondylolisthesis, lumbar spinal stenosis, and lumbar disc herniation. It has the advantages of mature technology, good clinical efficacy, and high fusion fixation efficiency (1). The anatomical structure of the spine is a spongy vertebral body, with a rich blood supply and a weak venous plexus. During PLIF, significant blood loss may occur, increasing the postoperative incidence rate and prolonging the clinical recovery time (2–4). In addition, most of the patients receiving PLIF are older adults, with poor hematopoietic function and extensive recovery time. Substantial blood loss is very likely to lead to serious complications such as myocardial infarction, cerebral infarction, pulmonary embolism, and severe anemia, requiring blood transfusion. Blood transfusion will not only increase the wound infection rate after surgery but may also cause many complications, such as hepatitis, AIDS, and other infectious diseases, as well as fever, vascular embolism, and delayed healing or even non-healing of wounds (5).

To reduce perioperative bleeding and avoid the adverse consequences of blood transfusion, antifibrinolytic drugs have been widely used (6), with tranexamic acid (TXA) being the most extensively used (7). TXA is an artificially synthesized amino acid derivative, the hemostatic effect of which is mainly related to the occupation of functional targets on fibrinolytic enzymes and fibrinogen (FIB). It stops the binding of fibrinolytic enzymes and fibrin, reduces the decomposition of fibrin, and ultimately plays a hemostatic role (8). Since the first application of TXA in total knee arthroplasty in 1995, extensive usage and significant research have confirmed that this drug can reduce perioperative blood loss and transfusion needs (9). However, we note that there is a pressing requirement for an efficient and safe alternative drug in clinical work due to temporary drug shortages of TXA in hospitals or its association with perioperative epilepsy and postoperative stroke during cardiac surgery (10–12). The mechanism of action of epsilon-aminocaproic acid (EACA) is similar to that of TXA. Although studies have shown (13) that TXA and EACA have similar hemostatic effects in the perioperative period of intertrochanteric fractures of the femur, there are significant differences between spinal surgery and limb surgery. Therefore, we adopted a retrospective cohort study to compare the clinical effects of intravenous application of EACA and TXA on reducing perioperative blood loss, observe the effectiveness and safety of the two drugs, and explore whether EACA can serve as a substitute for TXA, all during PLIF surgery.

## Materials and methods

### Inclusion and exclusion criteria

**Figure 1:**
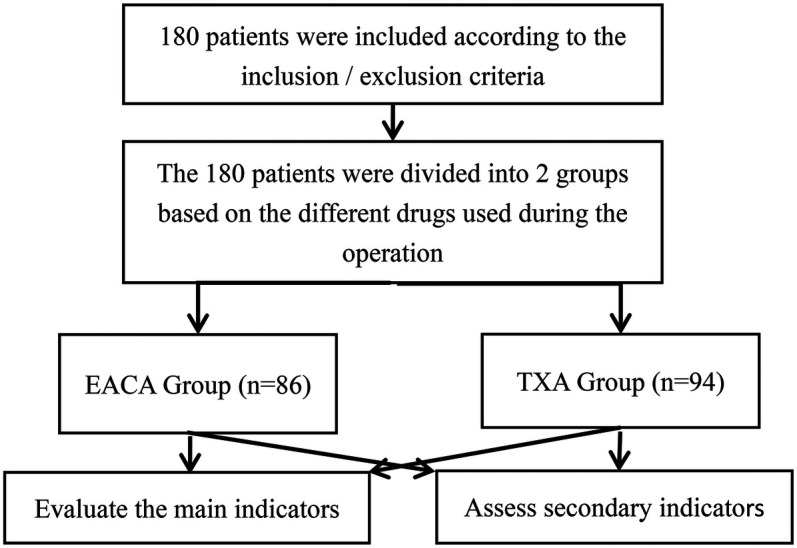
Patient flow chart.

Retrospective analysis was performed on patients with lumbar disc herniation, lumbar spinal stenosis, and lumbar spondylolisthesis who underwent PLIF and were admitted to the spinal surgery department of the Our hospital or The Sixth Affiliated Hospital of Xinjiang Medical University between September 2021 and May 2023 (Figure 1).

Inclusion criteria: (1) The included patients had to be over 18 years of age and with the ability to independently sign an informed consent form. (2) The patients were required to be diagnosed with lumbar disc herniation, lumbar spondylolisthesis, or lumbar spinal stenosis via CT and MRI examination, with indications for internal fixation fusion surgery. (3) The patients were required to have complete clinical data. Exclusion criteria: (1) Long-term use of anticoagulants, contraindications, and allergic reactions to tranexamic acid, aminocaproic acid, and perioperative medications were immediate criteria for exclusion. (2) Patients with preoperative anemia and hemoglobin (HB) levels of <12 g/dL for females and <13 g/dL for males were also rejected. (3) Patients with a history of thrombosis within the preceding 6 months were excluded from the analysis. (4) Finally, patients with abnormal preoperative coagulation function test results were also excluded. The implementation of this research plan complied with the Helsinki Declaration and was approved by the ethics committee of the Sixth Affiliated Hospital of Xinjiang Medical University. All research subjects signed informed consent forms.

### Surgical methods

Each patient was placed in a prone position for surgery. All surgeries were performed by the same anesthesia team with the patient under general anesthesia, with blood pressure controlled at 90–110/60–80 mmHg. After undergoing tracheal intubation and receiving general anesthesia, each patient was placed in a prone position. A midline incision was made at the back of the waist to strip off the paraspinal muscles. Conventional lumbar posterior fusion surgery was performed on the vertebrae, and pedicle screws were inserted in a standard manner. After nerve root decompression, the intervertebral space was filled with autologous bone, and the fusion cage was placed diagonally in the intervertebral space.

EACA group: This group received an intravenous infusion of 120 mg/kg EACA with 100 mL of physiological saline prior to the skin incision, followed by a slow intravenous infusion of the same dose and concentration of EACA every 6 h.

TXA group: This group was administered 1.0 g TXA via intravenous infusion with 100 mL of physiological saline before the skin incision was performed. After infusion, the same dose and concentration of TXA were slowly administered intravenously at an interval of 6 h.

**Figure 2:**
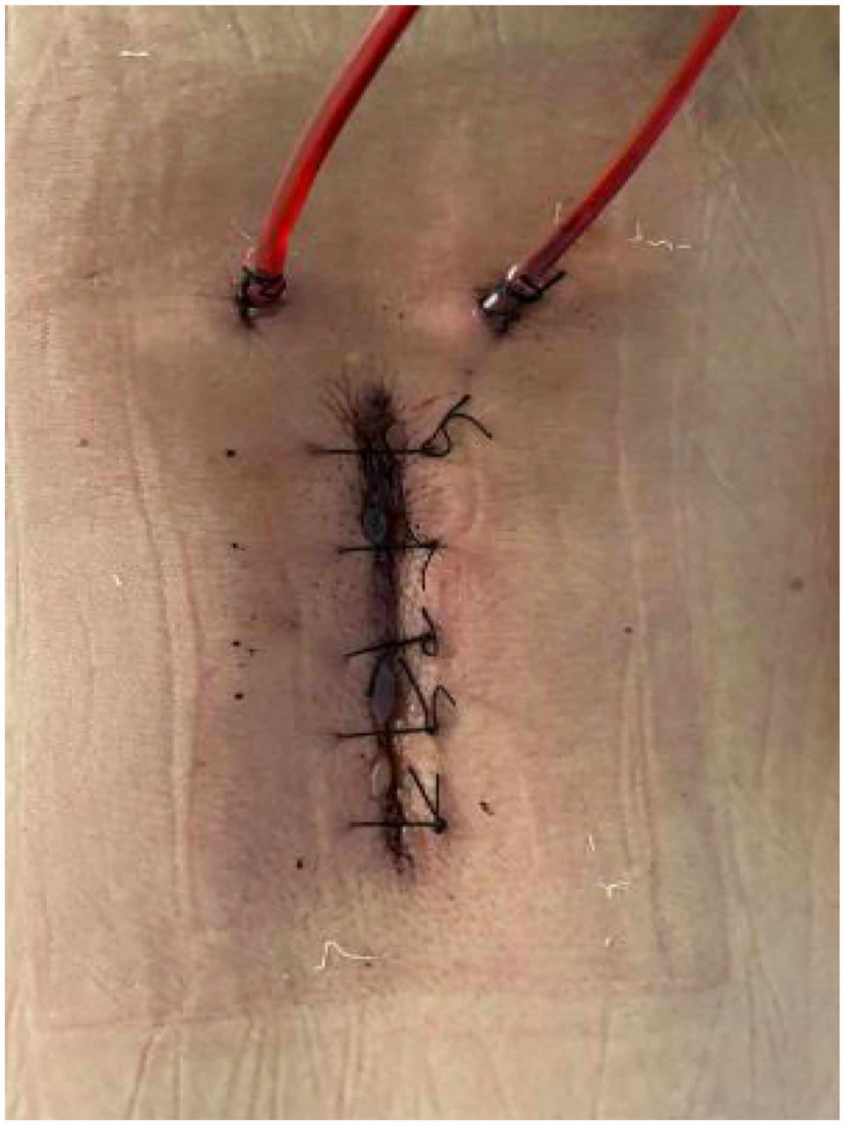
After surgery, all patients were fitted with two drainage pipes.

All patients were fitted with two negative pressure drainage tubes. In accordance with the “hematoma packing compression theory” (14), to prevent excessive bleeding, the drainage tubes were clamped for 4 h before opening. When the daily drainage volume was less than 30 mL, the drainage tubes were removed. The nurse measured the drainage volume using a measuring cup and recorded it (see Figure 2). The patients were then routinely transferred to the anesthesia recovery room after surgery and returned to the inpatient ward 1 h later.

### Postoperative management

All patients received the same postoperative treatment, including intravenous prophylactic use of antibiotics, mechanical prevention of venous thrombosis, and postoperative pain management. During hospitalization, the patients were asked to observe the condition of their lower limbs. If there was swelling in the lower limbs, a lower limb arteriovenous ultrasound examination was performed to confirm thrombosis. The patients' lower limb condition was evaluated during hospitalization and one month after surgery. If lower limb swelling was present, a lower limb arteriovenous ultrasound examination was performed. When pulmonary embolism was suspected, D-dimer and chest CT examination were immediately performed to confirm it. If the postoperative hemoglobin level was <70 g/L or ≥70 g/L but accompanied by symptoms such as dizziness, pale complexion, and weakness, blood transfusion was given. The hemoglobin levels were reevaluated 6 h after transfusion and the same criteria were utilized to reconsider transfusion.

### Outcome measurements

The following indicators were recorded: (1) Basic information: patient age, gender, body mass index (BMI), operation time, estimated blood volume, basic disease, and preoperative biochemical hematological indicators [red blood cells (RBC), HB, hematocrit (HCT), RBC width (SD), total protein (TP), albumin (ALB), globulin (GLB), serum ferritin (SF), D-dimer, FIB, fibrinogen degradation products (FDP), and international normalized ratio (INR)]. (2) Main outcome measures: total blood loss, total red blood cell loss, transfusion volume, and transfusion volume. (3) Secondary outcome measures: postoperative final biochemical hematological indicators [RBC, HB, HCT, RBC width (SD), TP, ALB, GLB, SF, D-dimer, FIB, FDP, and INR], length of hospital stay (LOH), total hospital expenses, and incidence of lower limb venous thrombosis.

Among the indicators, preoperative blood volume was calculated using the Gross equation (15)and Nadler equation method (16): Total blood volume (TBL) = k1 × height (m)^3^ + k2 × weight (kg) + k3, with male coefficients k1 = 0.366 9, k2 = 0.03219, k3 = 0.6041, female coefficients k1 = 0.3561, k2 = 0.033 08, k3 = 0.1833. Total RBC loss (L) = preoperative blood volume (L) × (preoperative HCT − postoperative HCT). Total bleeding volume = intraoperative bleeding volume + postoperative drainage volume. Intraoperative bleeding volume: The weight of postoperative gauze weighed + the amount of blood in the aspirator − the amount of flushing solution (30 mL can completely wet the small gauze, 180 mL can completely wet the large gauze, and the amount of physiological saline used for flushing is subtracted from the volume of the aspirator).

### Statistical processing

We used SPSS 26.0 software statistical process all data. We used case (rate) representation of count data by means of the χ^2^ test. The measurement data are expressed as mean ± standard deviation, and Student’s *t*-test is used. A value of *p* < 0.05 suggests that the differences are statistically significant. The statistical methods used in this study have been reviewed by biostatistics experts from Xinjiang Medical University.

## Results

### Patient demographics

The general preoperative information for the two groups of patients is shown in Table 1. The following indicators were included in both groups: age, gender composition, BMI, basic disease, and preoperative biochemical blood tests, including RBC, HB, HCT, RBC width (SD), TP, ALB, GLB, SF, D-dimer, and INR. The difference between fibrinogen and fibrinogen degradation products was not statistically significant (*p* > 0.05).

**Table 1:** Comparison of the two groups of basic data.

Index	EACA (*n* = 86)	TXA (*n* = 94)	*p*
Age (y, ±S)	67.10 ± 14.22	68.96 ± 13.56	0.372
Sex (*n*, male/female)	32/54	33/61	0.769
BMI (kg/m^2^, $\bar{\chi }\pm S$)	23.20 ± 3.90	23.80 ± .64	0.286
Basic disease (*n*)			
Hypertension	7	16	0.075
Diabetes	6	13	0.135
Osteoporosis	10	14	0.520
Preoperative values			
RBC (1,012/L)	3.88 ± 0.70	3.95 ± 0.69	0.498
HB (g/dL)	118.57 ± 22.50	120.22 ± 21.32	0.613
HCT (%)	35.61 ± 6.31	36.16 ± 6.82	0.580
RBC width (SD)	42.00 ± 10.15	40.05 ± 11.92	0.238
TP (g/L)	64.85 ± 8.04	66.27 ± 6.17	0.183
ALB (g/L)	39.61 ± 5.26	39.72 ± 4.99	0.886
GLB (g/L)	24.00 ± 3.73	24.35 ± 3.71	0.529
SF (μmol/L)	9.71 ± 10.07	11.04 ± 5.51	0.269
D-dimer (mg/L)	0.55 ± 0.41	0.54 ± 0.64	0.843
FIB (g/L)	3.85 ± 1.25	3.51 ± 1.08	0.056
FDP (mg/L)	1.43 ± 1.10	1.42 ± 1.26	0.920
INR	1.06 ± 0.18	1.08 ± 0.12	0.357

TXA, tranexamic acid; EACA, epsilon-aminocaproic acid; BMI, body mass index (weight/height^2^); RBC, red blood cell; HB, hemoglobin; HCT, hematocrit; TP, total protein; ALB, albumin; GLB, globulin; SF, serum ferritin; FIB, fibrinogen; FDP, fibrinogen degradation products; INR, international normalized ratio.

The continuous value is given as the mean and the standard deviation. Categorical values are given as the number of patients.

### Comparison of main outcome measures

The main outcome indicators and a comparison between the two groups of patients are shown in Table 2. Although the total blood loss in the EACA group was slightly higher (443.24 ± 149.93) than the total blood loss in the TXA group (429.17 ± 147.67), there was no statistically significant difference in the total blood loss between the two groups (*p* > 0.05). The total red blood cell loss in the EACA group (21.34 ± 24.86) and that in the TXA group (20.30 ± 25.80) were similar with was no statistically significant difference between the two groups (*p* > 0.05). The difference in blood transfusion volume and transfusion rate between the two groups of patients was not statistically significant (*p* > 0.05).

**Table 2:** Comparison of main outcome measures.

Index	EACA (*n* = 86)	TXA (*n* = 94)	*p*
PBV (L)	3.86 ± 0.62	4.03 ± 0.66	0.088
TBL (mL)	443.24 ± 149.93	429.17 ± 147.67	0.527
Total loss of RBC	21.34 ± 24.86	20.30 ± 25.80	0.784
Intraoperative bleeding volume (mL)	201.91 ± 92.38	184.38 ± 78.70	0.174
Postoperative drainage volume (mL)	241.33 ± 122.20	244.79 ± 128.25	0.853
Volume of transfusion (mL)	29.07 ± 114.63	38.30 ± 95.62	0.560
Transfusion (*n*, 100%)	8 (9.30%)	11 (11.70%)	0.601

PBV, patient's blood volume; TBL, total blood loss; RBC, red blood cell.

The continuous value is given as the mean and the standard deviation. Categorical values are given as the number of patients.

### Comparison of RBC, HB, HCT, and RBC width after surgery

**Figure 3:**
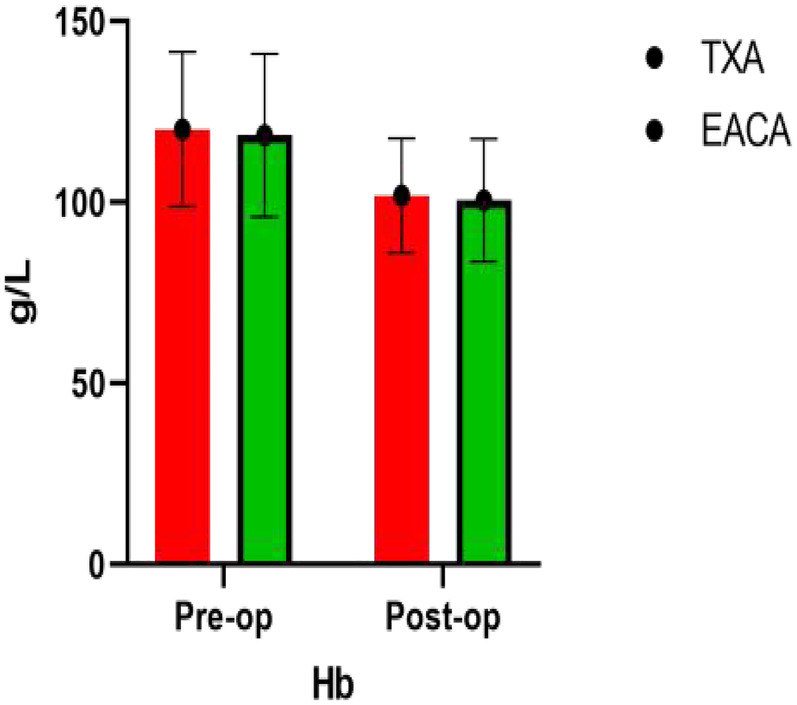
Comparative map of HB before and after operation.

The RBC width of the EACA group was significantly higher (43.94 ± 10.56) than the TXA group (40.45 ± 12.54), and the difference between the two groups was statistically significant (*p* < 0.05). However, there was no significant difference in other indexes such as RBC, HB, and HCT between the two groups. The comparison of hemoglobin before and after the operation is shown in Figure 3 with the results of the related data summarized in Table 3.

**Table 3:** Postoperative erythrocyte, HB, hematocrit, and erythrocyte width.

Variable	EACA (*n* = 86)	TXA (*n* = 94)	*p*
RBC (10^12^/L)	3.26 ± 0.55	3.33 ± 0.49	0.319
HB (g/dL)	100.59 ± 16.93	101.89 ± 15.75	0.595
HCT (%)	30.17 ± 4.74	31.19 ± 4.17	0.125
RBC width (SD)	43.94 ± 10.56	40.45 ± 12.54	0.044^a^

RBC, red blood cell; HB, hemoglobin; INR, international normalized ratio; FDP, fibrin degradation product.

The continuous value is given as the mean and the standard deviation.

a *p* < 0.05, and the differences between the two groups were statistically significant.

### Comparison of PT, ALB, GLB, and SF after operation

**Figure 4:**
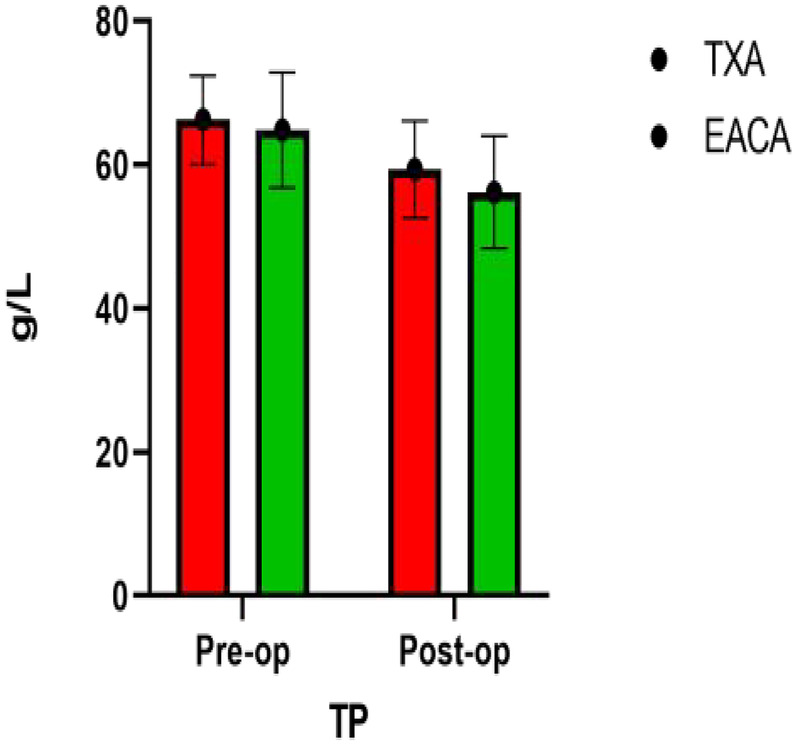
Comparative map of total protein before and after operation.

The postoperative total protein in the EACA group was 56.17 ± 7.83and that in the TXA group was 59.3628 ± 6.73. The postoperative TP in the TXA group was significantly higher than that in the EACA group. There was no significant difference in other indexes such as ALB, GLB, and SF between the two groups (*p* > 0.05). The comparison of total protein before and after the operation is shown in Figure 4 with the results of the related data summarized in Table 4.

**Table 4:** Postoperative TP, ALB, GLB, and SF.

Variable	EACA (*n* = 86)	TXA (*n* = 94)	*p*
TP (g/L)	56.17 ± 7.83	59.3628 ± 6.73	0.004^a^
ALB (g/L)	32.51 ± 4.35	33.63 ± 5.00	0.114
GLB (g/L)	22.52 ± 3.63	22.79 ± 3.75	0.633
SF (μg/L)	5.57 ± 5.12	6.80 ± 3.50	0.060

TP, total protein; ALB, albumin; GLB, globin; SF, serum ferritin.

The continuous value was given as the mean and the standard deviation.

a *p* < 0.05, and the differences between the two groups were statistically significant.

### Comparison of D-dimer, FIB, FDP, and INR after operation

**Figure 5:**
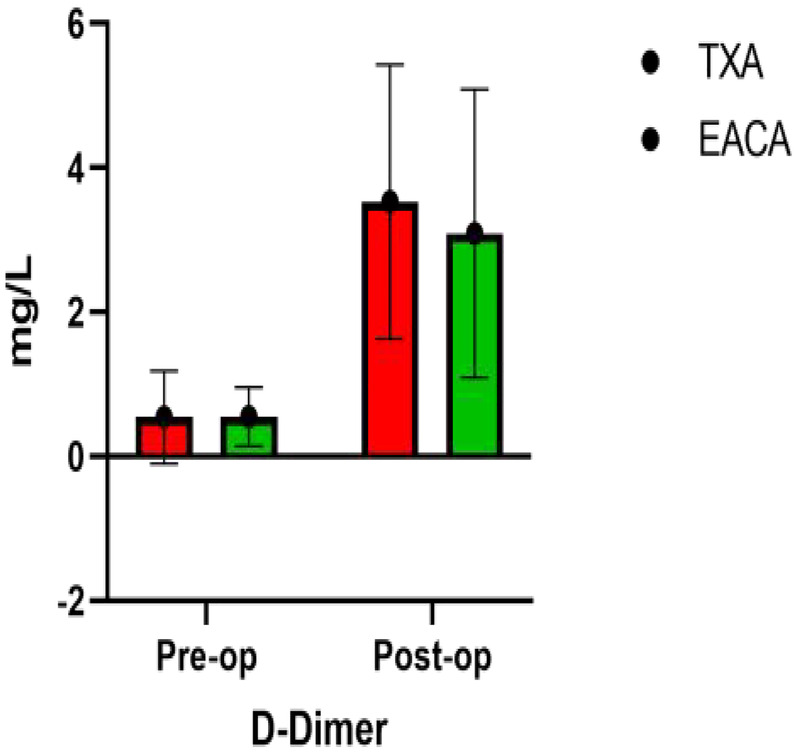
Comparative map of D-dimer before and after operation.

The postoperative INR of the EACA group and TXA group were 1.06 ± 0.14 and 1.14 ± 0.13, respectively. The postoperative INR of the TXA group was significantly higher than that of the EACA group. There was no significant difference in other indexes, such as D-dimer, fibrinogen, and fibrin degradation products, between the two groups (*p* > 0.05). The comparison of D-dimer before and after the operation is shown in Figure 5 with the results of the related data summarized in Table 5.

**Table 5:** Postoperative D-dimer, fibrinogen, fibrin degradation products, and INR.

Variable	EACA (*n* = 86)	TXA (*n* = 94)	*p*
D-dimer (mg/L)	3.09 ± 2.00	3.53 ± 1.90	0.135
FIB (g/L)	3.85 ± 1.25	3.51 ± 1.08	0.840
FDP (mg/L)	1.43 ± 1.10	1.42 ± 1.26	0.483
INR	1.06 ± 0.14	1.14 ± 0.13	0.000^a^

FIB, fibrinogen; FDP, fibrin degradation product; INR, international normalized ratio.

The continuous value was given as the mean and the standard deviation.

a *p* < 0.05, and the differences between the two groups were statistically significant.

### Comparison of length of stay, operative time, total hospital expense, and deep venous thrombosis of lower extremities

There was no significant difference in hospitalization time, operation time, total hospitalization cost, and deep venous thrombosis of lower extremities between the two groups (*p* > 0.05). The related results are summarized in Table 6.

**Table 6:** LOH, operative time, hospital costs, and thrombotic events.

Variable	EACA (*n* = 86)	TXA (*n* = 94)	*p*
LOH (day)	18.72 ± 4.93	18.84 ± 5.14	0.874
Operative time (min)	143.66 ± 38.67	150.64 ± 39.93	0.236
Expenses^a^	43,491.68 ± 13,223.09	45,980.55 ± 14,143.95	0.225
Thromboembolic event (*n*, 100%)	10 (11.62%)	17 (18.09%)	0.226

LOH, length of hospital stay.

The continuous value is given as the mean and the standard deviation.

a Results are presented in Chinese yuan.

## Discussion

PLIF is a common procedure for lumbar degenerative diseases; however, it is frequently associated with considerable perioperative blood loss (17). Wang et al. reported blood loss during PLIF to be as high as 1,260 mL, underscoring the significant perioperative risk posed by hemorrhage (18). TXA, a widely adopted antifibrinolytic agent and included in the WHO’s essential medicines list since 2011, has demonstrated efficacy in reducing surgical bleeding (19). However, its use is accompanied by concern regarding adverse effects, such as seizures, stroke, and allergic reactions (7). These risks highlight the need for safer alternative antifibrinolytic agents. EACA, which shares a similar mechanism of action as TXA but does not cross the blood–brain barrier, is associated with a lower risk of central nervous system-related adverse events, like seizures. Although EACA has an established record in cardiac surgery, its application in spinal surgery remains underexplored. Thus, the present study was designed to evaluate the hemostatic efficacy and safety of EACA in the context of PLIF.

Our findings indicate that intravenous usage of EACA and TXA yield comparable hemostatic outcomes. No significant differences were observed between the two groups in terms of total blood loss, total red blood cell loss, intraoperative bleeding, or postoperative drainage volume. When transfusion rate was considered as a clinical endpoint, 11 patients (11.70%) in the TXA group and 8 patients (9.30%) in the EACA group received transfusions, a difference that was not statistically significant (*p* > 0.05). This suggests that both agents are similarly effective in minimizing blood loss and transfusion requirements. EACA, a lysine analog antifibrinolytic, has been well studied in cardiac settings and is recognized for its favorable safety profile (20). Although TXA remains a first-line medication in many orthopedic applications, EACA has garnered increasing interest as a potential alternative for reducing blood loss in major orthopedic procedures (21).

Given the limited evidence regarding EACA's use in PLIF, we referred to studies within general orthopedic surgery. Our results align with previous comparative reports. For instance, Bradley et al. found no significant differences in hematologic outcomes, complications, or length of stay between patients receiving EACA and those receiving TXA in joint arthroplasty (22). Similarly, Riaz et al. reported comparable operative times and thromboembolic rates between the two agents (23). A randomized controlled trial involving 194 total knee arthroplasty patients noted that although calculated blood loss was higher in the EACA group, transfusion rates did not differ significantly between the EACA and TXA groups, reinforcing the notion of clinical comparability (24).

Orthopedic surgeries, including PLIF, are associated with non-negligible risks of deep vein thrombosis (DVT), attributable to surgical trauma, prolonged immobilization, and the use of antifibrinolytics, which may tilt the hemostatic balance toward a prothrombotic state (7). In our study, DVT was observed in 10 (11.62%) patients in the EACA group and 17 (18.09%) in the TXA group; this difference did not reach statistical significance (*p* = 0.226), suggesting a comparable safety profile in terms of thrombotic risk. This finding is consistent with that of Zhou et al., who also reported no significant difference in thrombotic events between the EACA and TXA groups (10). Nonetheless, the observed numerical increase in DVT events associated with TXA warrants attentive monitoring and further investigation.

Although our study demonstrated comparable hemostatic efficacy and a similar overall safety profile between EACA and TXA during PLIF surgery, it is important to acknowledge the specific safety concerns associated with EACA that are documented in the literature and drug labeling. EACA has been associated with upper urinary tract bleeding, which may lead to ureteral or renal pelvic clot obstruction and acute kidney injury—particularly when hematuria originates from the upper tract. Therefore, caution is advised in such scenarios, unless the benefits clearly outweigh the risks (25). In addition, cases of myopathy and rhabdomyolysis have been reported, which can progress to myoglobinuria and renal failure, especially with prolonged use. Monitoring of creatine kinase (CK) is recommended in prolonged therapies to mitigate this risk (26). Thromboembolic events, though rare, including intracardiac thrombosis and pulmonary embolism, have been observed, particularly in cardiac surgery settings (27). Rapid intravenous administration of EACA may also induce hypotension, bradycardia, or arrhythmias, underscoring the importance of slow infusion and adequate dilution (28). Other rare hematologic and neurologic adverse effects, such as agranulocytosis, leukopenia, seizures, and stroke, have been listed in labeling, though causality remains uncertain in many cases (29). These potential risks highlight the need for careful patient selection, appropriate dosing, and vigilant monitoring when using EACA, especially in populations with underlying renal or cardiac comorbidities.

Regarding the observed statistically significant differences in certain laboratory values—specifically red blood cell distribution width (RDW), TP, and INR—we interpret these findings with caution due to their limited clinical relevance. The slight elevation in postoperative INR in the TXA group (1.14 ± 0.13 vs. 1.06 ± 0.14 in the EACA group) and higher TP values (59.36 ± 6.73 vs. 56.17 ± 7.83 g/L) fell within normal physiologic ranges and did not correlate with clinical outcomes such as bleeding or thromboembolic events. Moreover, after adjusting for multiple comparisons using the Bonferroni method, these differences lost statistical significance, suggesting that they may represent random variations rather than true pharmacologic effects (8, 16). Although the incidence of thrombotic events was not statistically significant (11.62% in the EACA group vs. 18.09% in the TXA group, *p* = 0.226), the observed numerical trend warrants thoughtful consideration. This pattern is consistent with previous reports indicating that TXA, with its higher antifibrinolytic potency and longer half-life, may be associated with a modest increase in prothrombotic risk in certain surgical populations (10, 11). Therefore, while our study did not demonstrate a significant difference in thrombotic complications, the trend underscores the need for heightened clinical vigilance and further investigation through larger, adequately powered trials to thoroughly evaluate the safety profiles of both agents.

This study has several limitations that should be taken into consideration: (1) As a retrospective study, the results are susceptible to selection bias and unmeasured confounding factors. Although propensity score matching was applied to improve intergroup comparability and all analyses were adjusted for measurable baseline variables, the non-randomized design precludes causal inference. In addition, the lack of blinding may have influenced outcome assessment, particularly for subjective endpoints. Although we made every effort to ensure data completeness through rigorous extraction and validation from medical records, missing or inconsistently documented parameters cannot be entirely ruled out. (2) Although the Gross and Nadler equations are well validated and widely applied in estimating perioperative blood loss, they remain surrogate models rather than direct measurements. Thus, some degree of imprecision in quantitative blood loss assessment is inevitable. (3) The optimal dose and timing of EACA and TXA during spinal fusion surgery have not been standardized. Our results are based on a specific regimen, and the generalizability to alternative protocols requires further investigation. Consequently, the findings should be interpreted as exploratory, and validation through prospective randomized controlled trials is strongly recommended.

## Conclusions

Based on the findings of this retrospective study, intravenous EACA demonstrates comparable efficacy to TXA in reducing perioperative blood loss and transfusion requirements in patients undergoing posterior lumbar interbody fusion surgery. However, although not statistically significant, the numerically higher incidence of thrombotic events associated with TXA merits serious attention. These results suggest that EACA may represent a viable alternative to TXA in this surgical context, but broader clinical recommendations should await validation from larger, prospective, and ideally randomized controlled trials that are sufficiently powered to clarify the safety profile—particularly regarding thrombotic risk—of both antifibrinolytic agents.

## Data Availability

The raw data supporting the conclusions of this article will be made available by the authors, without undue reservation.
